# Intergenerational continuity of depressive symptoms: genetic and environmental pathways

**DOI:** 10.1017/S0033291725101633

**Published:** 2025-09-08

**Authors:** Marie C. Navarro, Marthe de Roo, Albertine J. Oldehinkel, Catharina A. Hartman, Tina Kretschmer

**Affiliations:** 1 Faculty of Behavioral and Social Sciences, Department of Pedagogy and Educational Sciences, University of Groningen, Groningen, The Netherlands; 2 Department of Psychiatry, University of Groningen, University Medical Center Groningen, Groningen, The Netherlands; 3 Interdisciplinary Center Psychopathology and Emotion Regulation (ICPE), University of Groningen, University Medical Center Groningen, Groningen, The Netherlands; 4 Institute of Psychology, Friedrich-Alexander University Erlangen-Nuremberg, Erlangen, Germany

**Keywords:** Intergenerational continuity, Depressive symptoms, Adolescence, Gene–environment interplay, Genetic nurture, Parental warmth

## Abstract

**Background:**

Depression runs in families, with both genetic and environmental mechanisms contributing to intergenerational continuity, though these mechanisms have often been studied separately. This study examined the interplay between genetic and environmental influences in the intergenerational continuity of depressive symptoms from parents to offspring.

**Methods:**

Using data from the Dutch TRAILS cohort (*n* = 2201), a prospective, genetically informed, multiple-generation study, we examined the association between parents’ self-reported depressive symptoms (reported at mean age of 41 years) and offspring depressive symptoms, self-reported nearly two decades later, in adulthood (mean age: 29 years). We assessed the role of genetic (polygenic scores for depressive symptoms in parents and offspring) and environmental mechanisms (parental warmth during adolescence) in explaining intergenerational continuity of depressive symptoms in separate and combined models.

**Results:**

Parents’ depressive symptoms, offspring genetic predisposition, and parental warmth were associated with an increased risk of depressive symptoms in offspring. In the combined model, parents’ genetic predisposition was linked to their own depressive symptoms, which were linked to lower parental warmth, which, in turn, was linked to higher depressive symptoms in offspring, after accounting for offspring genetic predisposition, sex, age, and socioeconomic status.

**Discussion:**

Both genetic and environmental mechanisms contribute to the intergenerational continuity of depressive symptoms independently and in interplay. Despite a significant effect, the influence of parental warmth was modest, suggesting limited covariation between this particular parenting measure and depressive symptoms, at least when assessed with large temporal distance.

## Introduction

Depression is one of the most prevalent mental health disorders and is projected to become the leading cause of global disease burden by 2030, according to the World Health Organization (World Health Organization, 2011). Depression is characterized by a set of symptoms, such as persistent sadness, lack of pleasure, interest, energy, or motivation, sleep disturbances, irritability, and withdrawal from friends and family, which collectively impair everyday functioning (Costello, 1993). Although depression can occur throughout the lifespan, adolescence and young adulthood are critical periods, as biological, emotional, and social transformations during these stages make youth particularly vulnerable to the onset of depression (Shorey, Ng, & Wong, 2022). Sex differences in depression also emerge during adolescence, with girls being at twice the risk of developing depression compared to boys (Hyde, Mezulis, & Abramson, 2008). Depression with an onset in adolescence often persists into adulthood and can have adverse long-term consequences, such as poor mental and physical health, lower educational achievement, or substance use problems (Bohman, Låftman, Alaie, Ssegonja, & Jonsson, 2024; Clayborne, Varin, & Colman, 2019).

‘Intergenerational continuity of depressive symptoms’ refers to the tendency for depressive symptoms to run in families. Offspring of parents with depressive symptoms are more likely to develop depressive symptoms themselves (Weissman et al., 2006), with both genetic and environmental pathways explaining transmission. Twin and adoption studies have demonstrated that depressive symptoms have a heritability of approximately 30–40%, meaning that genetic factors explain ~30–40% of the differences in depressive symptoms risk between individuals in a population (Flint & Kendler, 2014; Sullivan, Neale, & Kendler, 2000). Thus, shared genes are important in the transmission of depressive symptoms risk from one generation to another and offer an explanation for why depressive symptoms tend to run in families.

That said, environmental experiences are also implicated in the development of depressive symptoms (Yap, Pilkington, Ryan, & Jorm, 2014). For instance, childhood trauma, low socio-economic status, and harsh parenting practices have been linked to an increased risk for depressive symptoms. Conversely, parental warmth, defined as acceptance, caring, and positive support from parents toward their offspring (Rothenberg et al., 2020), is essential for promoting emotional security, attachment, and self-esteem in children. As such, low parental warmth can lead to emotional difficulties, negative beliefs, low self-esteem, and increased sensitivity to life stressors in offspring, resulting in a higher risk of developing depressive symptoms (National Research Council and Institute of Medicine, 2009). Of note, parents who suffer from depressive symptoms tend to show less warmth toward their offspring (Butterfield et al., 2021) and are more withdrawn, disengaged, and less likely to express affection toward their children (Lovejoy, Graczyk, O’Hare, & Neuman, 2000), which might contribute to the continuity of depressive symptoms across generations. However, parenting practices are not purely environmental influences, as they may themselves be shaped by parental genetic predispositions.

Indeed, genetic and environmental factors also act in interplay in contributing to the risk of depressive symptoms. In this study, we define the interplay between genes and environment as the dynamic ways in which genetic predispositions and environmental exposures influence each other and jointly contribute to the development of depressive symptoms. Genetic nurture, as one such interplay mechanism, refers to the idea that environmental factors that increase the risk for depressive symptoms may themselves be genetically influenced (Kong et al., 2018). In other words, the genes of parents, whether transmitted or not to offspring, influence the child’s environment, experiences, and parent–child interactions, which in turn shape the child’s development (Plomin & Bergeman, 1991; Scarr & McCartney, 1983; Wertz et al., 2019). As such, parental genetic risk acts both through the direct transmission of genetic risk to offspring and by shaping the environment in which children are raised.

### Limitations of previous research

Although research on the intergenerational continuity of depressive symptoms has expanded, genetic and environmental factors have often been considered separately. This is despite increasing interest in studying their interplay, probably because the latter remains methodologically challenging (Gotlib, Goodman, & Humphreys, 2020; Weissman, 2020). The first challenge lies in the nature of the data required for such research. To fully capture the continuity of depressive symptoms across generations, data from longitudinal cohorts spanning several decades and involving multiple family members (i.e. parents and offspring) are necessary. Instead, many studies rely on data collected retrospectively, where parents and/or offspring are asked to report past parenting or depressive symptoms from long ago. Due to the challenges in collecting long-term longitudinal data, studies often assess parental depressive symptoms during the child’s adolescence, the same period in which offspring symptoms are typically examined (e.g. Pearson et al., 2013; Rajyaguru, Kwong, Braithwaite, & Pearson, 2021). These studies thus compare depressive symptoms in middle-aged parents to depressive symptoms in adolescent offspring. Indeed, few studies have investigated depressive symptoms in both generations during comparable developmental periods. Furthermore, the majority of studies on the continuity of depressive symptoms in the general population are not genetically informed. Instead, parents’ depressive symptoms or personality traits (e.g. neuroticism) are often used as proxies for genetic risk (e.g., Arnau-Soler et al., 2018; Jami, Hammerschlag, Bartels, & Middeldorp, 2021; Kang et al., 2022; Mackin et al., 2022), although these are also environmentally influenced. Finally, studies have overlooked how genetic predispositions might act in interplay with environmental experiences, such as parenting practices, and how interplay mechanisms influence depressive symptoms risk. Overall, there is a need for more integrative approaches that combine multiple generations, genetic, and environmental factors to better understand the intergenerational continuity of depressive symptoms.

### Current study

We used data from a longitudinal genetically informed multiple-generation cohort, which offers a suitable framework to investigate the intergenerational continuity of depressive symptoms while overcoming several limitations of previous studies. First, we studied the association between parents and offspring depressive symptoms, measured at approximately age 41 in the parent generation and age 29 in the offspring generation, thus in comparable developmental periods. Next, we examined whether intergenerational continuity in depressive symptoms could be partly explained by genetic transmission as indexed by polygenic scores. We hypothesized that shared genes would account for at least part of the covariance in depressive symptoms observed between parents and offspring. Results from controlling for genetic transmission also inform about genetic nurture: if parental genes predict parental depressive symptoms and these, in turn, predict offspring depressive symptoms, all while accounting for the direct pathway from parent genes to offspring genes to offspring depressive symptoms, genetic nurture can be inferred.

Since we were also interested in whether parenting acts as environmental pathway, we examined whether parental warmth mediated the relationship between parents’ depressive symptoms and depressive symptoms in offspring, first in a model without genetic data, and then in a model that accounted for genetic transmission. While we conceptualized parenting as an environmental factor in this study, we acknowledge that parenting behaviors may themselves be influenced by genetic predispositions and modeled this link explicitly. We expected to find support for both genetic and environmental mechanisms but had no hypothesis regarding the relative strength of effects.

## Methods

### Participants

Data come from the TRacking Adolescents’ Individual Lives Survey (TRAILS), a prospective cohort study of Dutch adolescents that has been running for nearly 25 years by now. Participants were selected from 135 schools in five municipalities, in both urban and rural areas of the North of the Netherlands. From the *n* = 2935 preadolescents initially invited to participate, *N* = 2230 completed the first wave of data collection in 2001/2002. Participants were then assessed every 2–3 years (see cohort description and cohort update for details, Huisman et al., 2008; Oldehinkel et al., 2015). The present study used data from the first wave (T1), when the participants were on average 11 years, and from the seventh wave (T7), conducted in 2019, when they were on average 29 years. The protocol of TRAILS was approved by the Dutch Central Committee on Research Involving Human Subjects (CCMO) and the local Medical Ethics Review Board, and consent was obtained from all participants.

### Measures

*Parents’ (Generation_0_ = G_0_) depressive symptoms* were self-reported by one parent, most often the mother (97%), who reported on their own symptoms during the first wave of data collection, when the parent respondents were on average 41 years. Depressive symptoms were measured using the 21-item version of the Depression Anxiety Stress Scales (DASS-21) (Lovibond & Lovibond, 1995). DASS-21 was designed to measure the severity of symptoms across three related but distinct dimensions: depression, anxiety, and stress. In this study, we only used the 7-item depressive symptoms subscale (e.g., ‘I found it difficult to take the initiative to do something’; ‘I felt I had nothing to look forward to’; *α* = .80). Items referred to the past week and were answered on a 4-point Likert scale (0 = never; 1 = sometimes; 2 = often; 3 = usually). The responses were averaged to compute final scores, where a higher score indicates greater symptom severity.

*Participants’ (Generation_1_ = G_1_) depressive symptoms* at age 29 were measured with the Adult Self Report questionnaire (ASR), using the Withdrawal/depressive symptoms and Anxiety/depressive symptoms subscales (*r* = .67; *p* < .001), which were averaged to create a global depressive symptoms score. Depression subscales from the ASR include both DSM-based and empirically derived symptom scales designed to capture different facets of depression. The Withdrawal/depressive symptoms subscale included 9 items (e.g., ‘I do not have much energy’; ‘I am not interested in much’; *α* = .80) and the Anxiety/depressive symptoms contained 18 items (e.g., ‘I am too fearful or anxious’; ‘I am nervous or tense’; *α* = .93). Items referred to the past 6 months and were answered on a 3-point Likert scale (0 = not at all/not true; 1 = a little/sometimes; 2 = clearly/often). The responses were averaged to compute final scores, where a higher score suggests experiencing higher or more frequent symptoms of depression. The reliability and validity of the ASR subscales for assessing depressive symptoms in adults have been demonstrated in several studies (Achenbach & Rescorla, 2003; Gotham, Unruh, & Lord, 2014; Guerrero, Hoffmann, & Pulkki-Råback, 2020). Considering the self-reported nature of depressive symptoms, which may not correspond exactly to diagnostic criteria, we selected the ASR subscales as our primary outcome. However, we also conducted supplementary analyses using the DSM-based depressive problems scale as the measure of depressive symptoms in G_1_ (14 items, *α* = 0.87).

*Parental warmth* was reported by offspring at age 11 using the Egna Minnen Beträffande Uppfostran (EMBU), a validated scale designed to measure offspring (G_1_) perception of their parents’ (G_0_) emotional support, affection, encouragement, and attentiveness (Markus, Lindhout, Boer, Hoogendijk, & Arrindell, 2003). Participants responded to 18 items (e.g., ‘Do you feel that your father/mother loves you?’; ‘Does your father/mother ever hug you?’) using a 4-point Likert scale (1 = no, never; 2 = yes, sometimes; 3 = yes, often; 4 = yes, almost always). The responses were averaged to obtain final scores separately for mothers (*α* = .91) and fathers (*α* = .91). The two scores (*r* = .84; *p* < .001) were then averaged to obtain an aggregated score of parental warmth, with higher scores indicating greater perceived parental warmth.

Details on *DNA extraction and the genotyping* procedure preceding computation of polygenic scores are described in the Supplementary Material. Polygenic scores (PGS) for depressive symptoms in parents and offspring were derived using summary statistics from a genome-wide association study of self-reported depressive symptoms (*N* ~ 800,000) (Howard et al., 2019). The PGS were computed using LDPred2-auto, which estimates SNP-heritability (*h^2^*) and the proportion of causal variants (*p*) directly from the data, making the parameter tuning process on a validation set unnecessary (Privé, Albiñana, Arbel, Pasaniuc, & Vilhjálmsson, 2023). Consistent with previous work using TRAILS data (e.g., de Roo et al., 2023), we restricted the analysis to high-quality HapMap3+ variants, which offer comprehensive genome coverage. We used the linkage disequilibrium reference panel based on European individuals from the UK Biobank provided by the LDPred2 developers. The polygenic scores for depressive symptoms explained up to 0.5% of the variance in G_0_ depressive symptoms and up to 2.2% in G_1_. To account for population stratification, we regressed the original polygenic scores on 20 principal components. The resulting residuals, representing the variance in polygenic scores not explained by the principal components of genetic ancestry (used to control for population stratification), were used in all analyses.

All models were adjusted for sex (0 = male; 1 = female), age of G_0_ and G_1_, and familial socioeconomic status (SES), computed as the average of five standardized items: maternal and paternal education, maternal and paternal occupation, and household income (*α* = .84) (Kretschmer, Veenstra, Deković, & Oldehinkel, 2017; Veenstra, Lindenberg, Oldehinkel, De Winter, & Ormel, 2006).

### Statistical analysis

Analyses were performed in *RStudio* (version 4.2.0) using the *lavaan* package. We computed descriptive statistics and pairwise correlations between variables, followed by path analyses to examine genetic and environmental pathways as explanations for the relationship between G_0_ and G_1_ depressive symptoms. We adjusted for G_1_ sex, age, G_0_ age, and familial SES in all models.

**Figure 1.**
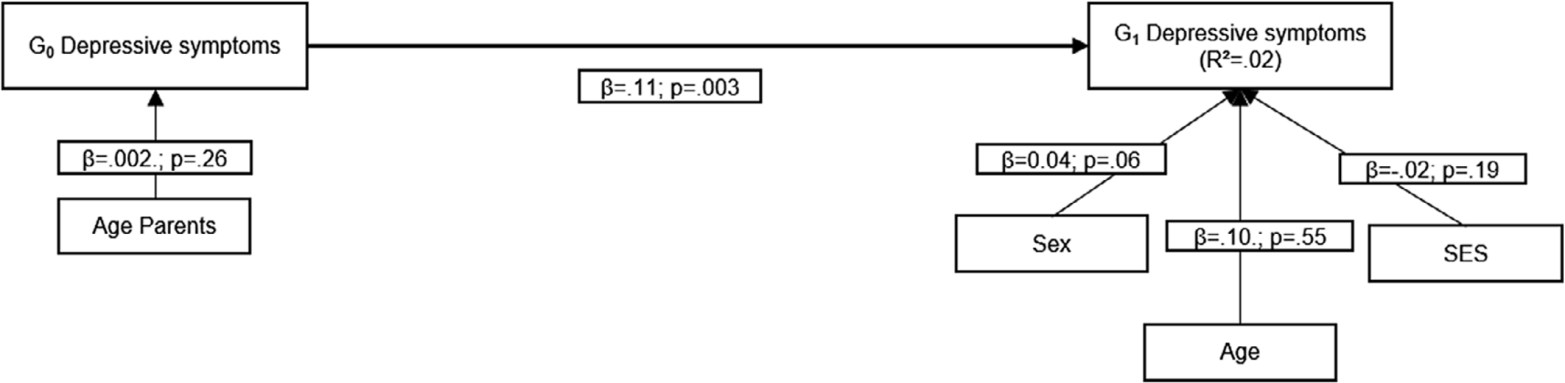
Association between G_0_ depressive symptoms and G_1_ depressive symptoms.

**Figure 2.**
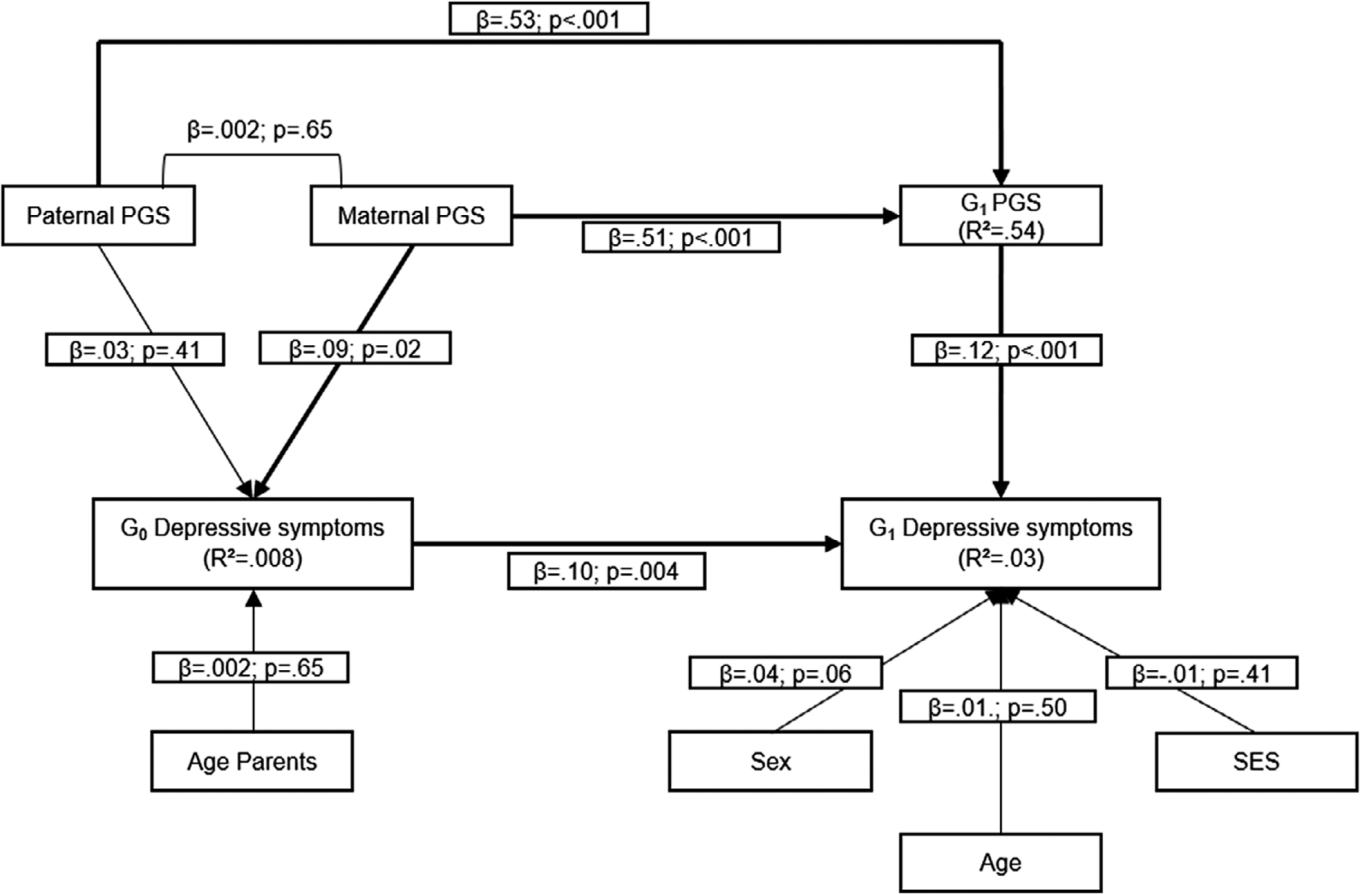
Intergenerational continuity of depressive symptoms, when genetic factors are involved (CFI = .90; RMSEA = .04).

**Figure 3.**
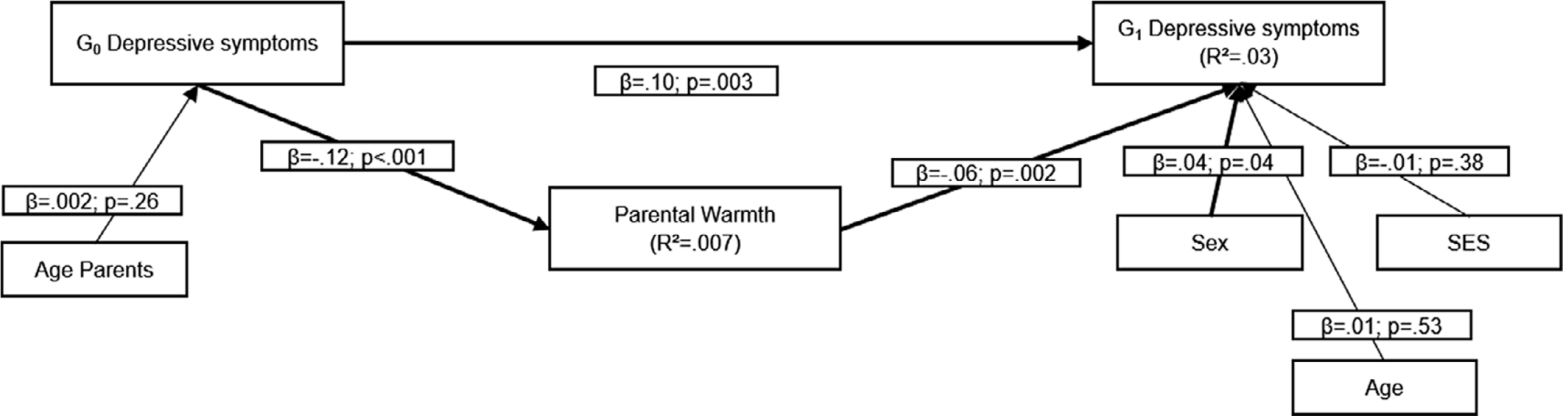
Association between G_0_ depressive symptoms and G_1_ depressive symptoms when mediated by parental warmth (CFI = .26; RMSEA = .08). Estimates of indirect and total effects were β = .007, p = .03 and β = .11, p = .001.

**Figure 4.**
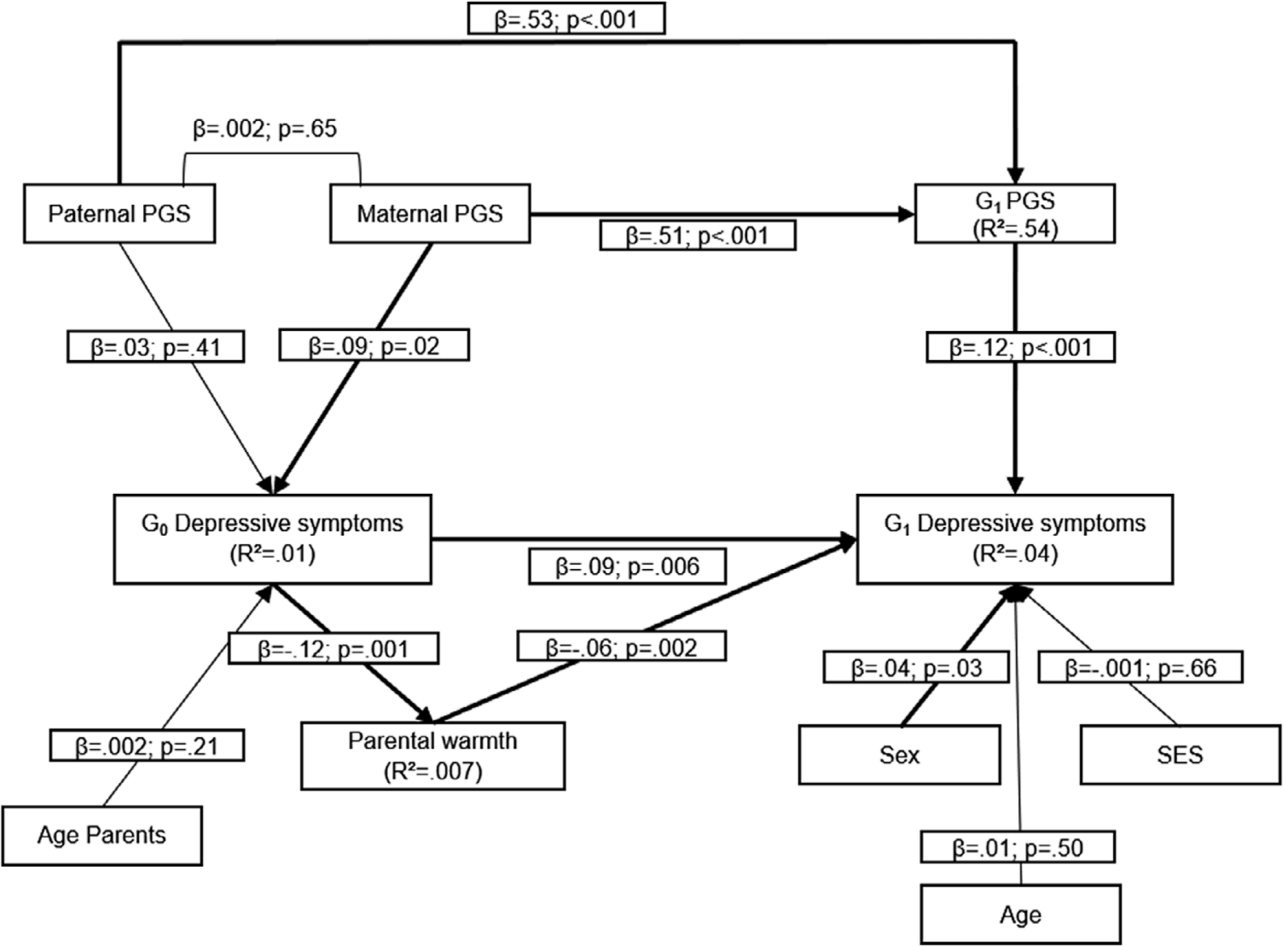
Genetic and environment interplay in the continuity of depressive symptoms (CFI = .81; RMSEA = .05). Estimates of indirect and total effects were β = .007, p = .03 and β = .10, p = .003.

First, we estimated the association between G_0_ depressive symptoms, reported at offspring age 11 and parent age ~ 41 years, and G_1_ depressive symptoms, reported at offspring age 29 years (Figure 1). Second, to examine shared genes as a potential explanation for intergenerational continuity of depressive symptoms from G_0_ to G_1_, we computed a model with a direct path between G_0_ depressive symptoms and G_1_ depressive symptoms, while G_0_ polygenic scores (paternal and maternal scores) predicted both G_0_ depressive symptoms and G_1_ polygenic score and G_1_ polygenic score predicted G_1_ depressive symptoms (Figure 2). Third, to examine environmental pathways, we computed a model where parental warmth was conceptualized as intermediate environmental factor between G_0_ depressive symptoms and G_1_ depressive symptoms (Figure 3). Fourth, in the final model, we included polygenic scores, for both G_0_ and G_1_, as well as parental warmth to study both pathways simultaneously (Figure 4).

To address missing data, we used full information maximum likelihood estimation, which resulted in a consistent sample size of *n* = 2201 for the models. We report standardized coefficients estimated over 1000 bootstraps. The use of bootstrap techniques is particularly recommended for the estimation of indirect effects, as their distribution is often non-normal. Bootstrapping accounts for this non-normality in the sampling distribution and improves the robustness of coefficient estimation (Preacher & Hayes, 2008). We used the Comparative Fit Index (CFI) and the Root Mean Square Error of Approximation (RMSEA) to evaluate the fit of the model, with CFI ≥ 0.95 and RMSEA ≤0.06 admitted as good fit. We also reported *R^2^* to indicate how much variance in the endogenous variables was explained by the explanatory variables.

## Results

Descriptive statistics of the sample and pairwise correlations are presented in Table 1. As expected, depressive symptoms in G_0_ were associated with depressive symptoms in G_1_, suggesting intergenerational continuity. Depressive symptoms in G_0_ were also more common in families with lower SES. Regarding genetic associations, both parents’ polygenic scores were positively correlated with offspring PGS, as one would expect, but only the mother’s polygenic score was significantly associated with G_0_ depressive symptoms. The offspring polygenic score was positively associated with both G_0_ and G_1_ depressive symptoms. Finally, parental warmth was significantly lower in G_0_ with more depressive symptoms, as well as in G_1_ with more depressive symptoms. Parental warmth was, however, higher in families with higher SES and for female offspring.

**Table 1.** Descriptive statistics and pairwise correlations of the variables used in this study

Variable	m	sd	Range	1. G_0_ Dep. symptoms	2. G_1_ Dep. Symptoms	3. Offspring PGS	4. Maternal PGS	5.Paternal PGS	6. Parental Warmth	7. Sex	8. SES	9. Age Parents
1. G_0_ Depressive symptoms	0.26	0.35	(0.00; 2.43)									
2. G_1_ Depressive symptoms	0.31	0.30	(0.00;1.58)	**.07 (.03)**								
3. Offspring PGS^a^				**.08 (.002)**	**.12 (<.001)**							
4. Maternal PGS^a^				**.12 (.001)**	.05 (.26)	**.50 (<.001)**						
5. Paternal PGS^a^				.03 (.51)	.07 (.12)	**.52 (<.001)**	.00 (.94)					
6. Parental warmth	3.22	0.50	(1.17; 4.00)	**−.10 (<.001)**	**−.10 (.001)**	−.02 (.42)	−.02 (.54)	.02 (.62)				
7. Sex	Male: 1086 (49.34%) Female: 1115 (50.65%)			.00 (.93)	**.09 (.002)**	.01 (.64)	−.06 (.08)	−.04 (.27)	**.10 (<.001)**			
8. SES^a^				**−.08 (<.001)**	−.02 (.52)	**−.15 (<.001)**	**−.07 (.03)**	**−.09 (.03)**	**.15 (<.001)**	.03 (.17)		
9. Age parents	40.60	4.59	(27.84; 57.98)	.04 (.10)	.01 (.78)	−.06 (.05)	−.08 (.03)	.02 (.62)	−.02 (.36)	.00 (.87)	**.34 (<.001)**	
10. Age offspring	11.11	0.56	(10.01;12.58)	−.02 (.43)	−.02 (.57)	.02 (.42)	−.01 (.67)	.04 (.32)	−.02 (.45)	−.04 (.10)	−.03 (.11)	**−.09 (<.001)**

Abbreviations: m, mean value; sd, standard deviation; Range, minimum and maximum value for every variable; PGS, polygenic scores; SES, familial socioeconomic status.

a Polygenic scores and SES were standardized (mean = 0, SD = 1), so their descriptive statistics are not presented in this table.

### Path models to study the continuity of depressive symptoms from G_0_ to G_1_

The first model estimated the association between both generations’ depressive symptoms without accounting for genetic or measured environmental factors. As expected, and already suggested by correlations, we identified a positive association between G_0_ depressive symptoms and G_1_ depressive symptoms, adjusting for familial SES, sex, and age (Figure 1; Supplementary Table 1).

In the model including both parents’ and offspring polygenic scores, G_0_ depressive symptoms remained significantly associated with G_1_ depressive symptoms. Genetic transmission was present, as G_0_ PGS were associated with G_1_ depressive symptoms via G_1_ PGS. We also identified a positive association between maternal PGS and G_0_ depressive symptoms, but paternal PGS was not associated with G_0_ depressive symptoms (Figure 2; Supplementary Table 1). In short, parents’ genes may influence offspring depressive symptoms both through genetic transmission and through their association with G_0_’s own depressive symptoms, which can also shape G_1_’s environment and are associated with greater risk for depressive symptoms in G_1_ as well.

When parental warmth was included as measured environmental mediator of the path from G_0_ depressive symptoms to G_1_ depressive symptoms, we found a negative association between G_0_ depressive symptoms and parental warmth, which in turn was negatively linked to G_1_ depressive symptoms. This indirect effect was also significant, indicating that intergenerational continuity of depressive symptoms is partly due to reduced parental warmth. The direct intergenerational continuity effect between G_0_ depressive symptoms and G_1_ depressive symptoms remained significant (Figure 3; Supplementary Table 1).

In the final model, in which both shared genes and parental warmth were tested as explanations for intergenerational continuity, environmental mediation via parental warmth remained significant with a negative association between G_0_ depressive symptoms and parental warmth, as well as from parental warmth to G_1_ depressive symptoms. In addition, G_1_ PGS remained significantly associated with their own depressive symptoms (Figure 4; Supplementary Table 1).

### Additional analyses

In a set of supplementary analyses, we repeated all models using the DSM-based depressive problems scale as the measure of depressive symptoms in G_1_ (Supplementary Figures 1–4). Overall, the pattern of results was consistent with those from the main analyses: we observed an association between depressive symptoms in G_0_ and depressive symptoms in G_1_, adjusting for SES, sex, and age. When including polygenic scores for both parents and offspring, the association between G_1_ PGS and their depressive symptoms as well as the association between G_0_ and G_1_ depressive symptoms remained significant. When parental warmth was added to the model, the overall pattern remained similar. Parents’ depressive symptoms were associated with lower levels of parental warmth, which in turn was negatively associated with offspring depressive symptoms, though the latter association, and the indirect effect, did not reach conventional levels of statistical significance (*p* = .07 and *p* = .12). The same pattern was observed in the fully adjusted model: G_0_ depressive symptoms were significantly associated with both parental warmth and G_1_ depressive symptoms, and although the association between parental warmth and G_1_ symptoms remained negative, it failed to meet the threshold for significance (*p* = .07). In all models, G_0_ PGS were significantly associated with G_1_ PGS, which in turn were associated with G_1_ depressive symptoms.

## Discussion

Using a genetically informed, longitudinal, multiple-generation cohort, we examined the continuity of depressive symptoms from parents to offspring and the contribution of genetic and environmental pathways, both independently and in combination, to this continuity. Genetic and environmental mechanisms partly explained this continuity, both in models in which these mechanisms were tested separately and in a combined model.

We found a positive association between G_0_ depressive symptoms, reported when offspring were 11 years old, and G_1_ depressive symptoms, reported nearly 20 years later, i.e. when offspring were in their late 20s or early 30s. Such intergenerational continuity aligns with prior research showing that offspring of depressed parents are at increased risk of developing depressive symptoms themselves (Weissman et al., 2016). That said, previous research has often examined associations between parents’ depressive symptoms and offspring depressive symptoms when both are assessed during adolescence, often due to the practical constraints of collecting long-term longitudinal data. We extend this research by showing intergenerational continuity of depressive symptoms when assessed in both parents and offspring during the same developmental period, adulthood, which has been less frequently studied.

In both generations, genetic risk as conceptualized by polygenic scores was linked to depressive symptoms, which validates the polygenic scores and is consistent with previous research (Kwong et al., 2021; Rice et al., 2019). That said, for the parent generation, only the mother’s polygenic score, but not the father’s, was associated with depressive symptoms. This probably reflects the fact that depressive symptoms in G_0_ were self-reported by a single parent, mainly the mother, so depressive symptoms mainly reflect mothers’ experiences. Given this imbalance, we conducted an additional set of analyses using only mothers’ information on depressive symptoms, polygenic scores, and parenting practices (Supplementary Figures 4–8). The results were consistent with the main findings, with very similar estimates, directions of association, and percentages of variance explained, which is expected given that the main analyses were primarily influenced by mothers’ information. The effect sizes for the associations between polygenic scores and depressive symptoms were similarly modest in both generations, and adding the genetic pathway to the model did not substantially alter the effect size of the association between G_0_ and G_1_ depressive symptoms, suggesting that parent–offspring similarity in depressive symptoms is only for a small part due to genetic risk, at least when captured by polygenic scores. Indeed, it is important to note that current polygenic scores explain only a small proportion of the total genetic variance in depressive symptoms, and more generally across complex traits or diseases, which likely contributes to the modest associations observed in our models (Dudbridge, 2013).

We also tested environmental pathways beyond parents’ depressive symptoms, as we were interested in concrete aspects of parenting that might convey risk. Parental warmth contributes to a supportive and secure family environment and contributes to children’s emotional development. A lack of parental warmth can increase children’s vulnerability to depressive symptoms (Butterfield et al., 2021; del Barrio, Holgado-Tello, & Carrasco, 2016). Indeed, the mediation model involving parental warmth showed that parents with more depressive symptoms tended to display lower levels of warmth. In turn, at least in the main analyses, lower parental warmth was associated with more depressive symptoms in offspring (Clayborne et al., 2021). While several studies have examined the link between parenting and offspring depression, much of this research has focused on earlier developmental periods or relied on cross-sectional designs (Goodman, Simon, Shamblaw, & Kim, 2020). By assessing parental warmth during early adolescence and depressive symptoms in offspring in adulthood, our study complements previous work by highlighting an association between early parenting behaviors and later mental health, suggesting that the implications of caregiving may extend beyond adolescence.

Overall, the explanatory power of our models was modest, with the full model, in which genetic and environmental factors were considered simultaneously, accounting for 4% of the variance in offspring depressive symptoms. Across all models, the proportion of explained variance ranged from 2% to 4%. These small effect sizes are consistent with findings from previous studies on the etiology of depressive symptoms in the general population and highlight the complex, multifactorial origins of depression. Indeed, no single factor, genetic, familial, or environmental, has so far independently explained a large proportion of individual differences in depressive symptoms. Within our models, parental warmth explained approximately 1% of the variance in offspring depressive symptoms, after accounting for parents’ depressive symptoms, genetic risk, and demographic factors. This small effect size is also consistent with previous studies and may be explained by the fact that parental warmth, or any single aspect of parenting, although important, represents only one of many factors that influence mental health later in life (Clayborne et al., 2021; McLeod, Weisz, & Wood, 2007). As a result, studying its specific contribution in isolation may result in a modest effect, as observed here. In the case of our study, another aspect to consider is the substantial time gap – nearly two decades – between assessments of parental warmth and offspring depressive symptoms. During that time, individuals are likely exposed to a plethora of intra- and extrafamilial experiences that can increase or reduce the risk for depressive symptoms. Finally, genetic risk in our study accounted for approximately 1% of the variance in offspring depressive symptoms, a result comparable to findings from other studies using polygenic scores as proxies for genetic inheritance (Musliner et al., 2015; Rabinowitz et al., 2020). These uniformly small effect sizes highlight the complexity of the etiology of depressive symptoms, involving the interplay of many small genetic and environmental influences rather than a single dominant one.

Most studies on the intergenerational continuity of depressive symptoms have examined genetic transmission and environmental effects separately (Collishaw et al., 2016; Kwong et al., 2021; Rice, 2010; Saluja et al., 2004). To overcome this limitation, we estimated genetic and environmental mechanisms in a combined model to explore their relative contributions to explaining the association between G_0_ and G_1_ depressive symptoms. Both pathways remained significant when studied together, suggesting that genetic predispositions and parenting practices contribute jointly, although modestly, to intergenerational continuity. These findings underscore the importance of integrative models that account for both genetic and environmental factors simultaneously, not only to estimate their independent contributions but also to test how these factors may interact and influence each other in shaping intergenerational risk for depressive symptoms (Jami et al., 2021; Lesch, 2004).

Supplementary analyses using the DSM-based depressive problems scale as depressive symptoms measure for G_1_ showed a comparable pattern of results. The main difference was that, although G_0_ depressive symptoms remained significantly associated with parental warmth, the association between parental warmth and offspring depressive symptoms did not reach conventional significance (*p* = .07). However, the direction and magnitude of the estimated effects were similar to those observed in the main analyses. The attenuation may reflect differences in how the scales capture depressive symptomatology. While the DSM-based scale includes a narrower set of symptoms aligned with diagnostic criteria, the empirically derived subscales may be more sensitive to environmental variation, such as parenting, particularly in a non-clinical sample like ours (de Wolff, Vogels, & Reijneveld, 2014; Dingle et al., 2011). In general though, the supplementary results support our overall conclusions.

Although the design of our study does not allow for causal conclusions or direct implications for intervention, our findings are in line with existing work on similarity in psychopathology across generations, suggesting that preventive efforts should prioritize improving parental mental health to limit the intergenerational continuity of depressive symptoms. Screening for depressive symptoms in parents could help identify families that might benefit from additional support, ensuring that parents struggling with depressive symptoms receive targeted interventions. Given the small effect size of parental warmth in our study, it is difficult to draw conclusions supporting interventions targeting parenting practices. However, other studies have suggested that early family-based interventions that promote positive parenting strategies, emotional support, and effective communication, especially for families where parents’ depressive symptoms are present, may further help in reducing the risk for psychopathology in offspring (Compas et al., 2011; Sandler et al., 2003). That said, both parental psychopathology and parenting explained only a small amount of variance in the outcome, which might be owed to the fact that many years and experiences lay between both assessments, which might affect psychopathology risk more strongly.

## Limitations and future directions

Despite several strengths in this study, including longitudinal data spanning two decades, the availability of genetic information for both parents, and the assessment of depressive symptoms in both generations during adulthood, some limitations need to be considered. First, while we had polygenic scores for depressive symptoms available for both parents, G_0_ depressive symptoms were self-reported by only one parent, most often the mother, so G_0_ depressive symptoms primarily reflect maternal experiences. Maternal and paternal depressive symptoms may not only influence intergenerational continuity in distinct ways, but may also impact parenting practices in different ways (Manuele, Yap, Lin, Pozzi, & Whittle, 2023). To address this limitation, we conducted supplementary analyses focusing only on mothers’ depressive symptoms, polygenic scores, and parental warmth, which supported the main findings (see Supplementary materials). Future studies should thus aim to assess depressive symptoms in both mothers *and* fathers directly from the person.

Second, we combined two subscales of the Adult Self Report questionnaire to measure offspring depressive symptoms. We chose a different approach than studies that combine the three ASR subscales, Withdrawal/depressive symptoms, Anxiety/depressive symptoms, and Somatic complaints for the following reasons: (a) the correlation between the withdrawal/depressive symptoms and anxiety/depressive symptoms subscales was high (~.70), suggesting substantial overlap and supporting their combination into a single measure; (b) we focused on affective and cognitive symptoms of depression, rather than somatic complaints, which may be influenced by other physical health issues and thus reduce measurement specificity for depression. Although somatic symptoms can be part of depression, some studies suggest they may represent a distinct construct (Ziebold et al., 2019); (c) the items in the somatic complaints subscale did not align with the items used to measure depressive symptoms in parents; and (d) we used polygenic risk scores for depressive symptoms, which do not align with somatic complaints or the broader concept of internalizing problems. Besides, neither the ASR nor DSM-based scales used to measure depressive symptoms in G_1_ fully align with the measure of depressive symptoms used for the parent generation. Ideally, future studies should use consistent measures of depressive symptoms across generations to ensure consistency and comparability.

Third, we focused on parental warmth as an environmental factor known to be influenced by parents’ depressive symptoms and important for offspring development. However, it is important to note that we relied on offspring-reported parental warmth, which may be subject to reporter bias and thus influence the observed associations (Taber, 2010). In addition, other environmental factors, both within and beyond parenting practices (e.g. family cohesion, violence, stressful life events), may also contribute to the intergenerational continuity of depressive symptoms. Future studies should explore how these additional factors may be influenced by parents’ mental health and, in turn, influence offspring well-being.

Fourth, we examined one type of gene–environment interplay, but other forms should also be explored. To this end, we conducted two linear regression analyses including polygenic scores for both parents and offspring, parental warmth, and parents’ depressive symptoms. In one model, we tested interaction terms between parents’ PGS and parental warmth; in the other, we tested interaction terms between parents’ PGS and parents’ depressive symptoms. Although the interaction effects were not statistically significant, these analyses are included in the supplementary materials (Supplementary Tables 3 and 4). Further mechanisms of gene–environment interplay are also important to consider in future research, such as evocative gene–environment correlation, in which offspring genetic predispositions influence parenting behaviors. In the context of depressive symptoms, offspring emotional reactivity or behavioral difficulties – both genetically influenced and associated with depressive symptoms – might influence parental feelings and behaviors, which might thus form a pathway from offspring genes to offspring outcomes. In our study, offspring polygenic scores were indeed correlated with parents’ depressive symptoms. However, as our analytical approach was not intended to formally test evocative gene–environment correlation and our explicit focus was on pathways through which parent genes affect offspring outcomes, we hope that future research can explore mechanisms driven by child genes.

## Conclusion

Our study suggests that both genetic and environmental mechanisms contribute, independently and in interplay, to the intergenerational continuity of depressive symptoms. Although genetic and environmental effects were modest, it remains important to further investigate family-related factors and potential interplay between them, as they may be interesting targets for interventions and offer opportunities to reduce risk across generations.

## Supporting information

Navarro et al. supplementary material Navarro et al. supplementary material

## Supplementary material

The supplementary material for this article can be found at http://doi.org/10.1017/S0033291725101633.
