# Supplementary material for: Intergenerational continuity of depressive symptoms: genetic and environmental pathways
Source: Psychol Med. 2025 Sep 8;55:e263. doi: 10.1017/S0033291725101633 (PMC13040585; doi:10.1017/S0033291725101633)
Supplement: Navarro et al. supplementary material [file S0033291725101633sup001.pdf]

SUPPLEMENTARY MATERIAL

Intergenerational continuity of depressive symptoms: Genetic and environmental pathways

Marie C. Navarro, Marthe de Roo, Albertine J. Oldehinkel, Catharina A. Hartman, Tina Kretschmer

Supp Methods1. Description of DNA extraction and participant exclusion.

Supp Table1. Parameter estimates, standard errors, and p-values for all path models included in the main analyses.

Supp Table2. Descriptive statistics and pairwise correlations of the variables used in this study, with DSM-based scale for depressive symptoms in G_1

Supp Table3 Coefficients from linear regression testing gene x environment interaction between parents' PGS and parental warmth.

Supp Table4. Coefficients from linear regression testing gene x environment interaction between parents' PGS and parents' depressive symptoms.

Supp Figure1. Association between G_0 depressive symptoms and G_1 depressive symptoms, measured with DSM-based scale.

Supp Figure2. Intergenerational continuity of depressive symptoms, measured with DSM-based scale in G_1, when genetic factors are involved.

Supp Figure3. Association between G_0 depressive symptoms and G_1 depressive symptoms, measured with DSM-based scale, when mediated by parental warmth.

Supp Figure4. Genetic and environmental interplay in the continuity of depressive symptoms, with G_1 depressive symptoms measured with DSM-based scale.

Supp Figure5. Association between mothers' depressive symptoms and G_1 depressive symptoms.

Supp Figure6. Intergenerational continuity of depressive symptoms between mothers and offspring, when genetic factors are involved.

Supp Figure7. Association between mothers' depressive symptoms and G_1 depressive symptoms, when mediated by maternal warmth.

Supp Figure8. Genetic and environmental interplay in the continuity of depressive symptoms between mothers and offspring.

Supp Methods1. Description of DNA extraction, genotyping, and participant exclusion.

Blood samples of participants in our sample were collected during the third wave of data collection, in 2006-2007, resulting in a sample of 1360 participants for genetic data. Those who did not provide blood samples were asked to provide buccal cells using Cytobrush® (n = 360). DNA extraction was performed using a manual salting-out method and stored at -80°C (Miller, Dykes, & Polesky, 1988). Genotyping followed the manufacturer's protocols and was conducted using the Golden Gate Illumina BeadStation 500 and Infinium™ HumanCytoSNP-12 v2.1 BeadChip platforms (Illumina Inc., San Diego, CA). DNA samples were excluded if they were highly heterogeneous, duplicated, related, or identified as non-European through principal component analysis. Additionally, genetic variants with more than 5% missing data, a minor allele frequency below 1%, or significant deviation from Hardy-Weinberg equilibrium ($p < 10^{-6}$) were removed. Genotype imputation was performed using the Haplotype Reference Consortium's global reference panel on the Michigan Imputation Server (Das et al., 2016; McCarthy et al., 2016). We only kept one member of each sibling pair; therefore, we excluded 28 participants, retaining the sibling with the most complete data or, in cases of ties, the one with the higher subject ID. Among the 1347 participants with genetic data, the analytic sample included 564 complete trios (mother, father, and participants), 804 mother–child complete duo, and 661 complete father–child duo.

The genetic data of the parents were analyzed a decade later than the genetic data of the TRAILS participants. As a result, the genetic data of both generations were analyzed using different genotyping chips and slightly different quality control procedures, leading to differences in available SNPs between parents and offspring. To ensure comparability across generations, we restricted our analyses to the SNPs that were both available for both parents and offspring with equal imputation quality. This approach minimizes potential biases due to differences in genotyping platforms and quality control procedures.

References:

Das, S., Forer, L., Schönherr, S., Sidore, C., Locke, A. E., Kwong, A., … Fuchsberger, C. (2016). Next-generation genotype imputation service and methods. *Nature Genetics*, *48*(10), 1284–1287. https://doi.org/10.1038/ng.3656

McCarthy, S., Das, S., Kretzschmar, W., Delaneau, O., Wood, A. R., Teumer, A., … Haplotype Reference Consortium. (2016). A reference panel of 64,976 haplotypes for genotype imputation. *Nature Genetics*, *48*(10), 1279–1283. https://doi.org/10.1038/ng.3643

Miller, S. A., Dykes, D. D., & Polesky, H. F. (1988). A simple salting out procedure for extracting DNA from human nucleated cells. *Nucleic Acids Research*, *16*(3), 1215. https://doi.org/10.1093/nar/16.3.1215

Supp Table 1. Parameter estimates, standard errors, and p-values for all path models included in the main analyses.

Outcome	Exp. variable	Estimate	SE	p
**Model 1**				
Dep G_1 ~	Dep G_0	.11	.04	.003
	SES	-.02	.01	.19
	Sex	.04	.02	.06
	Age	.10	.02	.55
Dep G_0 ~	Age Parents	.002	.002	.26
**Model 2**				
Dep G_1 ~	Dep G_0	.10	.03	.004
	SES	-.01	.01	.41
	Sex	.04	.02	.06
	Age	.01	.02	.50
	G_1 PGS	.12	.03	<.001
Dep G_0 ~	Age Parents	.002	.002	.65
	Paternal PGS	.03	.04	.41
	Maternal PGS	.09	.04	.02
G_1 PGS ~	Paternal PGS	.53	.02	<.001
	Maternal PGS	.51	.02	<.001
**Model 3**				
Dep G_1 ~	Dep G_0	.10	.03	.003
	SES	-.01	.01	.38
	Sex	.04	.02	.04
	Age	.01	.02	.53
	Parental warmth	-.06	.02	.002
Dep G_0 ~	Age Parents	.002	.002	.26
Parental warmth ~	Dep G_0	-.12	.03	<.001
**Model 4**				
Dep G_1 ~	Dep G_0	.09	.03	.006

Outcome	Exp. variable	Estimate	SE	p
	SES	-.001	.01	.66
	Sex	.04	.02	.03
	Age	.01	.02	.50
	G_1 PGS	.12	.03	<.001
	Parental warmth	-.06	.02	.002
Dep G_0 ~	Age Parents	.002	.002	.21
	Paternal PGS	.03	.04	.41
	Maternal PGS	.09	.04	.02
G_1 PGS ~	Paternal PGS	.53	.02	<.001
	Maternal PGS	.51	.02	<.001
Parental warmth ~	Dep G_0	-.12	.03	<.001

Supp Table 2. Descriptive statistics and pairwise correlations of the variables used in this study, with DSM-based scale for depressive symptoms in G_1

Variable	m	sd	Range	1. G_0 Dep. symptoms	2. G_1 Dep. symptoms	3.Offspring PGS	4.Maternal PGS	5.Paternal PGS	6.Parental Warmth	7.Sex	8. SES	9. Age Parents
1. G_0 Dep. symptoms	0.26	0.35	(0.00; 2.43)									
2. G_1 Dep. symptoms	0.31	0.30	(0.00 ;1.58)	**.09 (.003)**								
3. Offspring PGS*				**.08 (.002)**	**.14 (<.001)**							
4. Maternal PGS*				**.12 (.001)**	**.11 (.01)**	**.50 (<.001)**						
5. Paternal PGS*				.03 (.51)	.09 (.05)	**.52 (<.001)**	.00 (.94)					
6. Parental Warmth	3.22	0.50	(1.17; 4.00)	**-.10 (<.001)**	-.05 (.08)	-.02 (.42)	-.02 (.54)	.02 (.62)				
7. Sex	Male: 1086 (49.34%)<br>Female: 1115 (50.65%)			.00 (.93)	**.18 (<.001)**	.01 (.64)	-.06 (.08)	-.04 (.27)	**.10 (<.001)**			
8. SES*				**-.08 (<.001)**	-.03 (.25)	**-.15 (<.001)**	**-.07 (.03)**	**-.09 (.03)**	**.15 (<.001)**	.03 (.17)		
9. Age Parents	40.60	4.59	(27.84; 57.98)	.04 (.10)	.01 (.93)	-.06 (.05)	-.08 (.03)	.02 (.62)	-.02 (.36)	.00 (.87)	**.34 (<.001)**	
10. Age Offspring	11.11	0.56	(10.01;12.58)	-.02 (.43)	-.02 (.44)	.02 (.42)	-.01 (.67)	.04 (.32)	-.02 (.45)	-.04 (.10)	-.03 (.11)	**-.09 (<.001)**

m: mean value; sd: standard deviation; Range: minimum and maximum value for every variable; PGS = Polygenic scores; SES = Familial socioeconomic status.

*Polygenic scores and SES were standardized (mean = 0, SD = 1), so their descriptive statistics are not presented in this table.

Supp Table 3. Coefficients from linear regression testing gene x environment interaction between parents' PGS and parental warmth.

	β	SE	*p*
G_0 depressive symptoms	.11	.04	<.001
Paternal PGS	-.09	.23	.71
Maternal PGS	.10	.29	.73
Offspring PGS	.10	.06	.08
Parental Warmth	-.06	.02	.004
SES	-.006	.01	.57
Sex	.04	.02	.01
Offspring Age	.01	.02	.43
Paternal PGS x Parental Warmth	.02	.07	.72
Maternal PGS x Parental Warmth	-.03	.08	.69

Supp Table 4. Coefficients from linear regression testing gene x environment interaction between parents' PGS and parents' depressive symptoms.

	β	SE	*p*
G_0 depressive symptoms	.11	.04	.003
Paternal PGS	-.04	.08	.58
Maternal PGS	-.02	.06	.78
Offspring PGS	.12	.06	.05
Parental Warmth	-.06	.02	.01
SES	-.004	.01	.76
Sex	.04	.02	.07
Offspring Age	.01	.02	.50
Paternal PGS x G_0 depressive symptoms	.06	.10	.53
Maternal PGS x G_0 depressive symptoms	.02	.09	.80

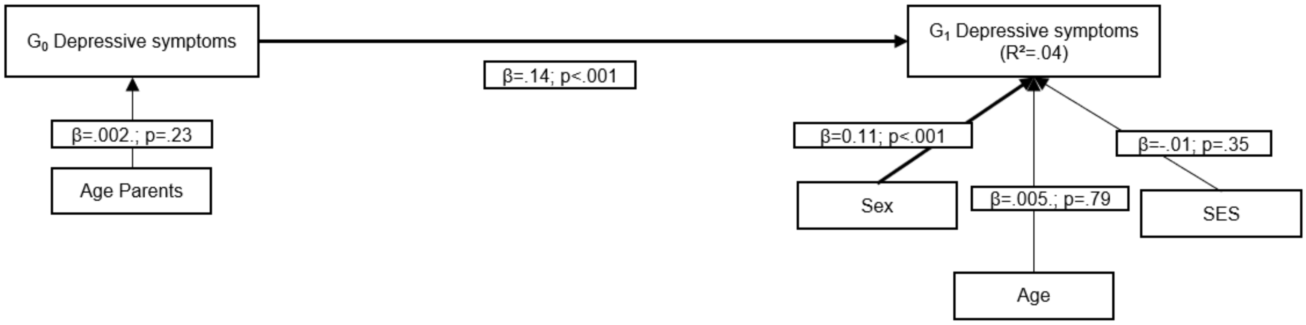

Supp Figure 1. Association between G_0 depressive symptoms and G_1 depressive symptoms, measured with DSM-based scale.

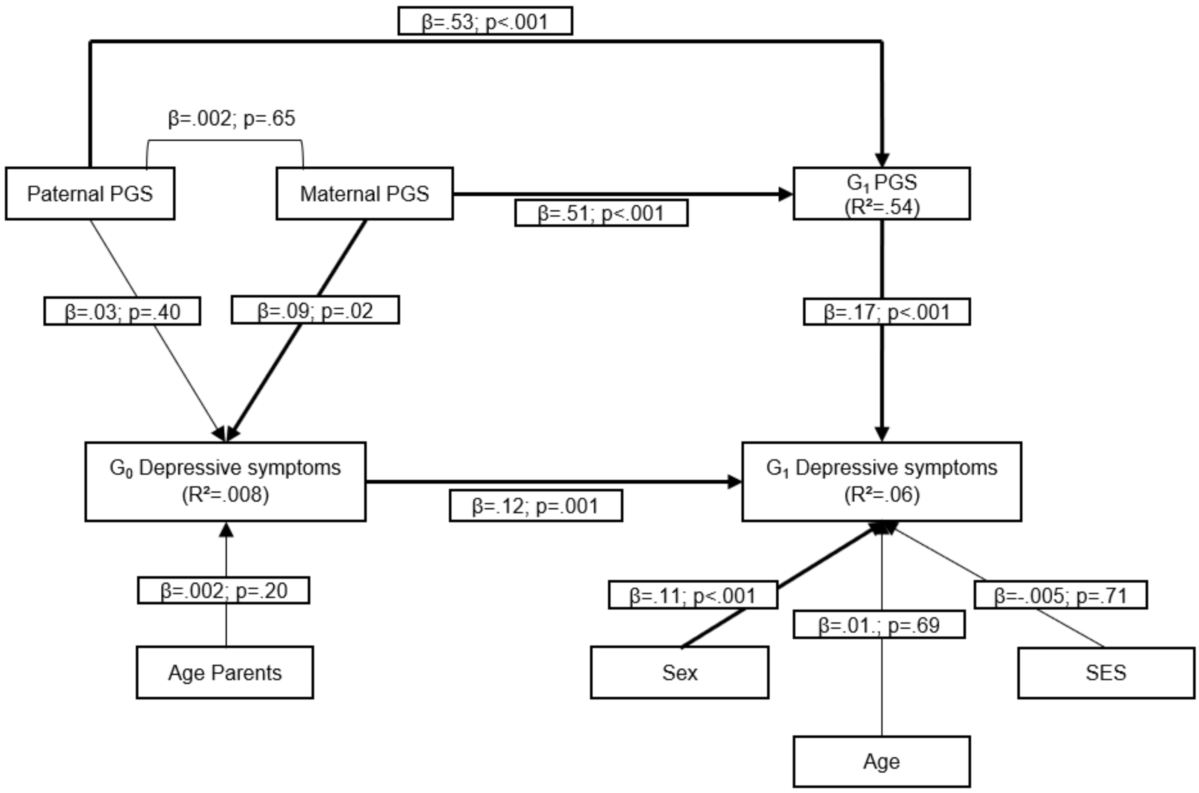

Supp Figure 2. Intergenerational continuity of depressive symptoms, with G_1 depressive symptoms measured with the DSM-based scale, when genetic factors are involved (CFI = .90; RMSEA = .04).

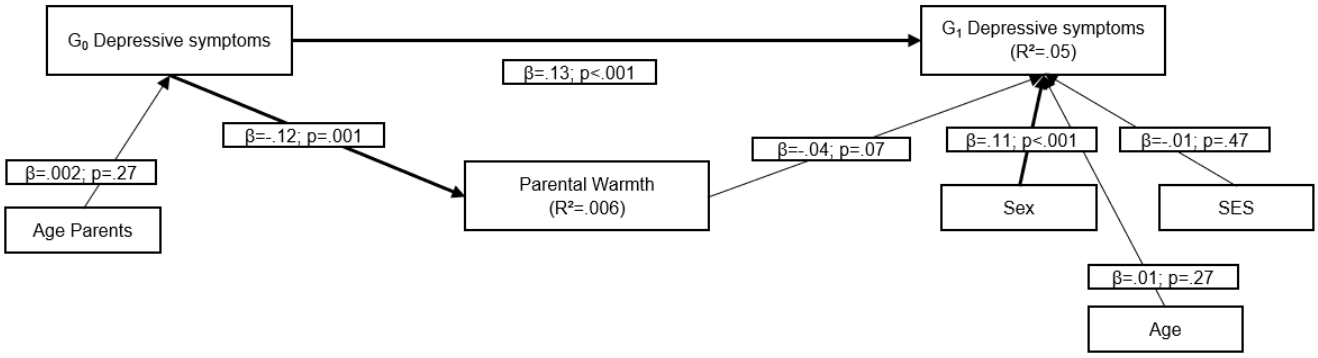

Supp Figure 3. Association between G_0 depressive symptoms and G_1 depressive symptoms, measured with the DSM-based scale, when mediated by parental warmth (CFI = .33; RMSEA = .08). Estimates of indirect and total effects were β = .005, p = .12 and β = .14, p < .001

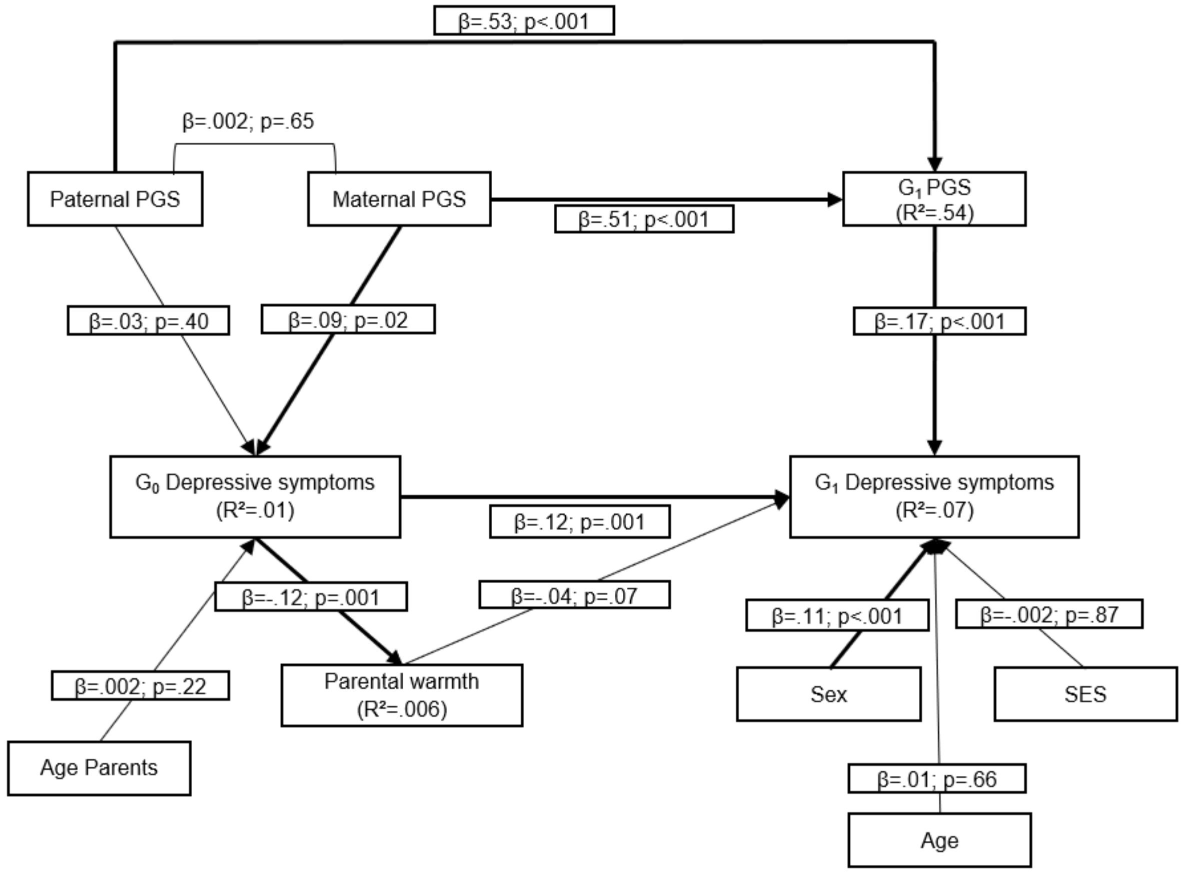

Supp Figure 4. Genetic and environment interplay in the continuity of depressive symptoms, measured with the DSM-based scale in G_1 (CFI = .82; RMSEA = .05). Estimates of indirect and total effects were β = .005, p = .12 and β = .12, p = .001

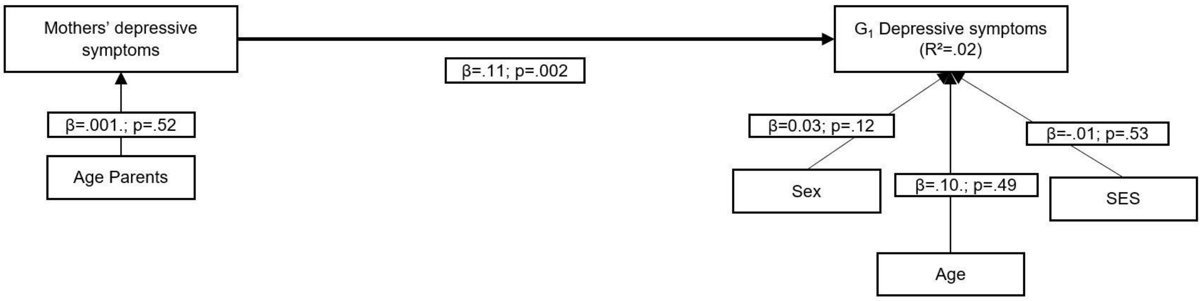

Supp Figure5. Association between mothers' depressive symptoms and G_1 depressive symptoms.

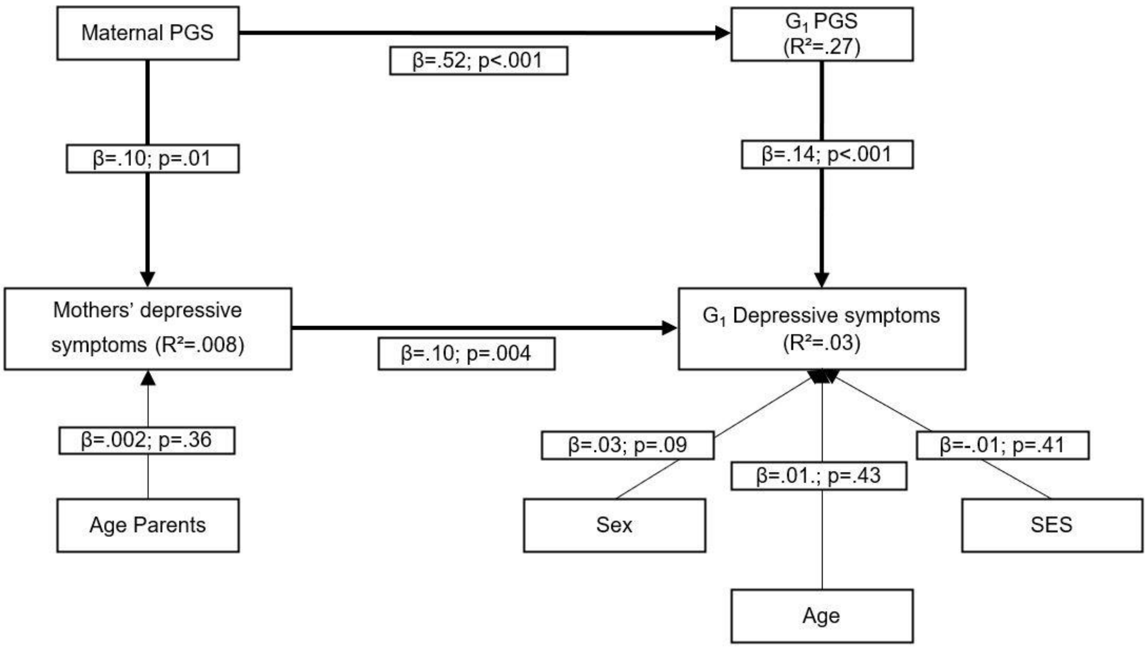

Supp Figure6. Intergenerational continuity of depressive symptoms between mothers and offspring, when genetic factors are involved.

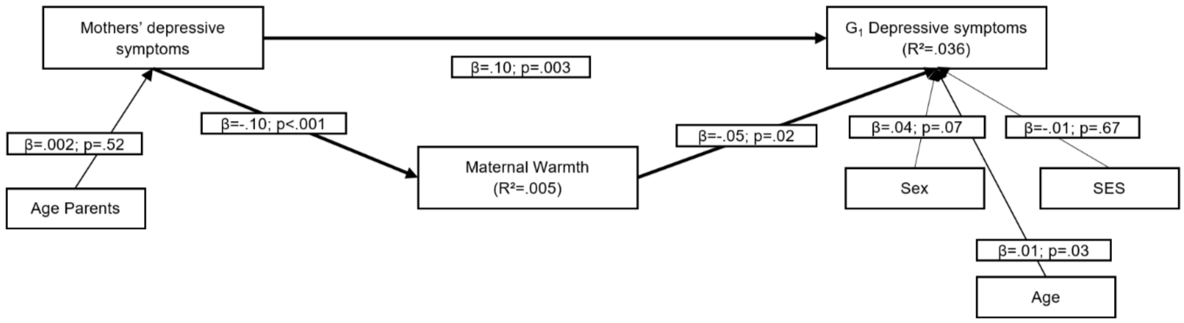

Supp Figure7. Association between mothers' depressive symptoms and G_1 depressive symptoms, when mediated by maternal warmth.

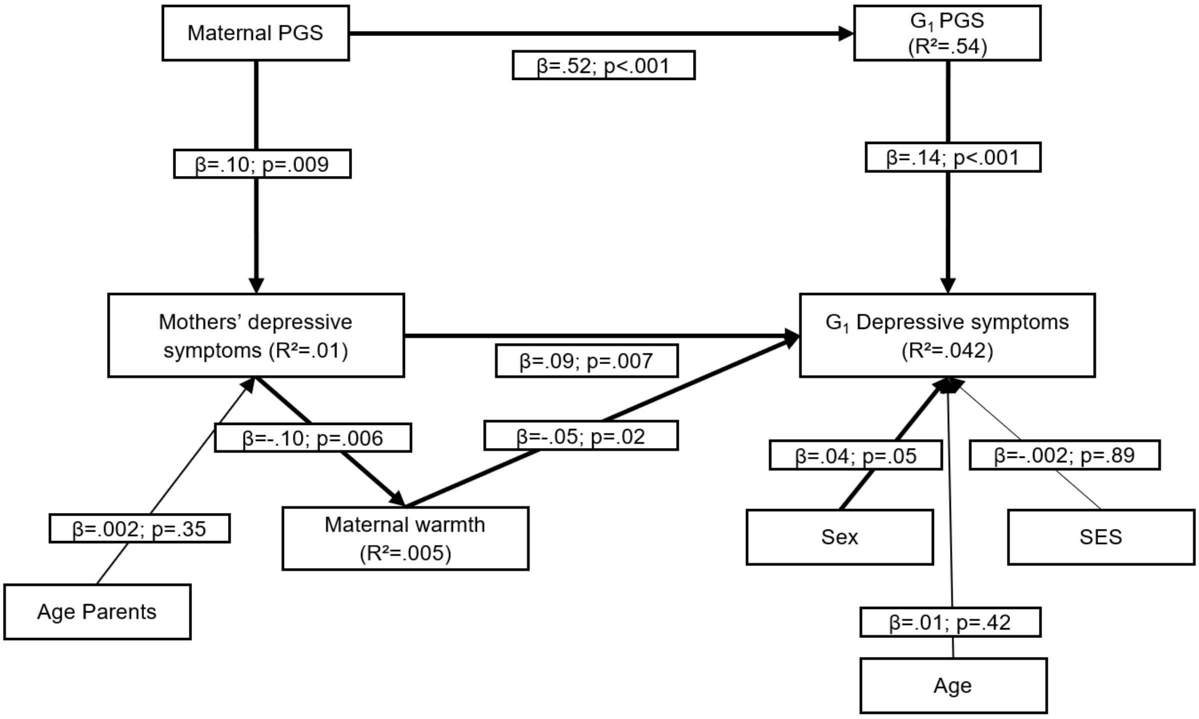

Supp Figure8. Genetic and environmental interplay in the continuity of depressive symptoms between mothers and offspring.
